# Deciphering the Pharmacological Mechanisms of Ma Xing Shi Gan Decoction against COVID-19 through Integrating Network Pharmacology and Experimental Exploration

**DOI:** 10.3389/fphar.2020.581691

**Published:** 2020-11-26

**Authors:** Qianqian Li, Chen Bai, Ruocong Yang, Weiying Xing, Xiaohan Pang, Siying Wu, Shaoyang Liu, Jianxin Chen, Tiegang Liu, Xiaohong Gu

**Affiliations:** School of Traditional Chinese Medicine, Beijing University of Chinese Medicine, Beijing, China

**Keywords:** Ma Xing Shi Gan decoction, COVID-19, Traditional Chinese Medicine, immunomodulatory, antiviral

## Abstract

The outbreak of new infectious pneumonia caused by SARS-CoV-2 has posed a significant threat to public health, but specific medicines and vaccines are still being developed. Traditional Chinese medicine (TCM) has thousands of years of experience in facing the epidemic disease, such as influenza and viral pneumonia. In this study, we revealed the efficacy and pharmacological mechanism of Ma Xing Shi Gan (MXSG) Decoction against COVID-19. First, we used liquid chromatography–electrospray ionization tandem mass spectrometry (LC-ESI-MS/MS) to analyze the chemical components in MXSG and identified a total of 97 components from MXSG. Then, the intervention pathway of MXSG based on these components was analyzed with network pharmacology, and it was found that the pathways related to the virus infection process were enriched in some of MXSG component targets. Simultaneously, through literature research, it was preliminarily determined that MXSG, which is an essential prescription for treating COVID-19, shared the feature of antiviral, improving clinical symptoms, regulating immune inflammation, and inhibiting lung injury. The regulatory mechanisms associated with its treatment of COVID-19 were proposed. That MXSG might directly inhibit the adsorption and replication of SARS-CoV-2 at the viral entry step. Besides, MXSG might play a critical role in inflammation and immune regulatory, that is, to prevent cytokine storm and relieve lung injury through toll-like receptors signaling pathway. Next, in this study, the regulatory effect of MXSG on inflammatory lung injury was validated through transcriptome results. In summary, MXSG is a relatively active and safe treatment for influenza and viral pneumonia, and its therapeutic effect may be attributed to its antiviral and anti-inflammatory effects.

**Graphical Abstract:**
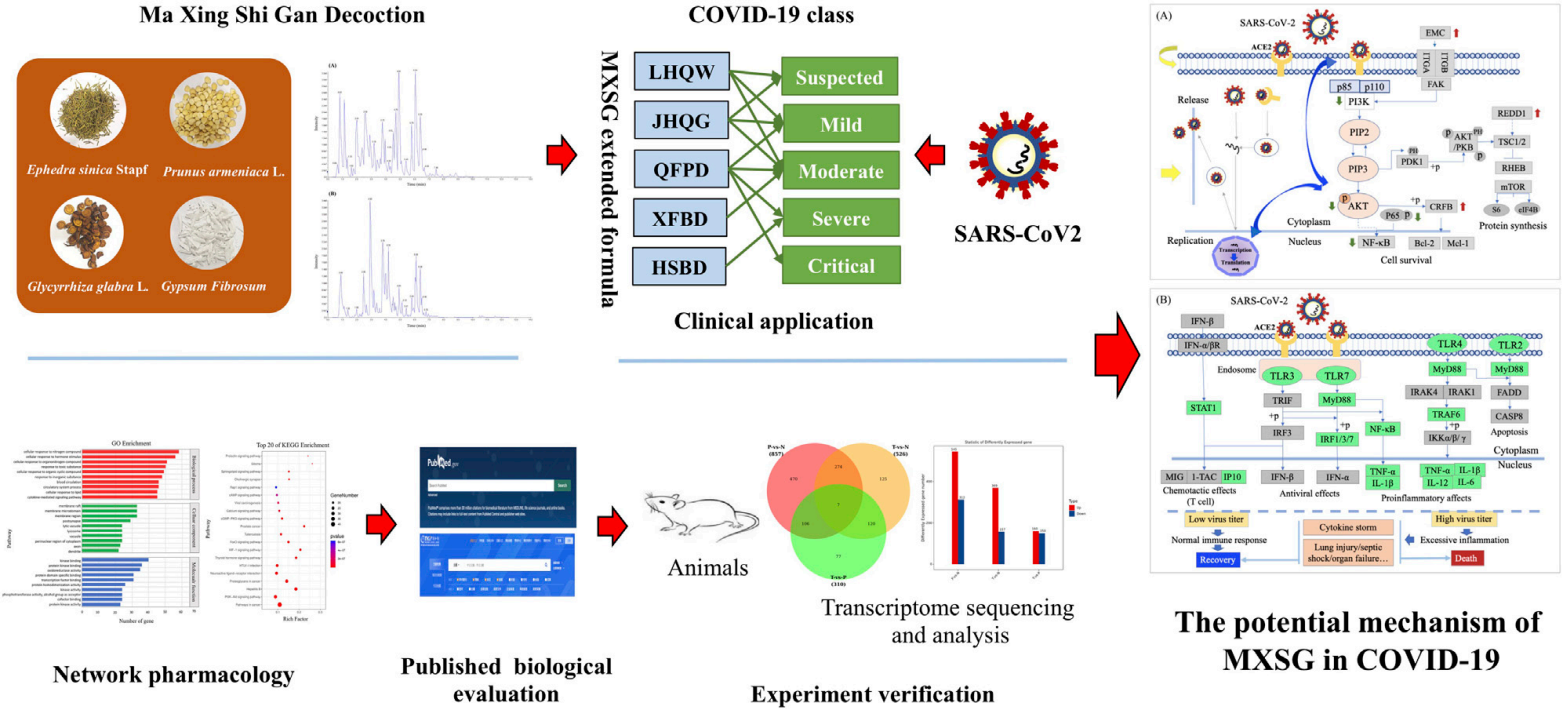
Study design:the workflow for the whole study

## Introduction

The coronavirus disease 2019 (COVID-19) epidemic is a global public health crisis, with considerable mortality and morbidity exerting pressure on health care and the economy. Unfortunately, there are currently no drugs or vaccines available to treat specific antivirals (Zumla et al., 2016; Xie et al., 2020). The majority of patients infected with SARS-CoV-2 show symptoms of pneumonia, fever, dry cough, fatigue, and other symptoms such as myalgia, and diarrhea (Henry and Vikse, 2020; Stower, 2020; Xiong et al., 2020). Like SARS-CoV, the spine (S) protein of SARS-CoV-2 enters human alveolar epithelial cells by binding the angiotensin-converting enzyme 2 (ACE2) receptor (Zhou et al., 2020). At low viral titers, the human immune response may be characterized by the antiviral response to type I interferon and the CD4^+^ and CD8^+^ T-cell response, leading to viral clearance. Severe infection and excessive immune-inflammatory response caused by high viral titer have been proved to be the leading cause of progression to acute lung injury (ALI), acute respiratory distress syndrome, respiratory and circulatory failure, and even death (Chen et al., 2020; Narasaraju et al., 2020; Wang et al., 2020). Patients with COVID-19 exhibit pathogenesis; clinical manifestations are similar to the symptoms of SARS-CoV and H1N1 infections. Therefore, although the pathogenesis of COVID-19 is poorly understood, the similar mechanisms of SARS-CoV and H1N1 can give us a great deal of information on the pathogenesis of SARS-CoV-2 infection to promote our recognition (Li et al., 2020c).

Based on clinical observation of COVID-19 and experience in treating SARS and H1N1, the guideline on diagnosis and treatment of COVID-19 has proposed that combines modern medicine with TCM in China (Liu et al., 2012; Li and Peng, 2013; Dei-Cas et al., 2020). According to the Press Conference of the Joint Prevention and Control Mechanism of State Council, 74,187 people have used Chinese medicine in confirmed cases of COVID-19 in China. It accounted for 91.5% of the total cases. Clinical observation shows that the total effective rate of TCM has reached more than 90% (National Health Commission of the People’s Republic of China, 2020). Ma Xing Shi Gan (MXSG) decoction, was the basic formula of three drugs and three formulas including Lian Hua Qing Wen capsules (LHQW), Jin Hua Qing Gan granules (JHQG), Qing Fei Pai Du decoction (QFPD), Hua Shi Bai Du decoction (HSBD), and Xuan Fei Bai Du decoction (XFBD) promulgated by China’s National Health Commission for the treatment of COVID-19. It has been applied to COVID-19 patients in both suspected cases and confirmed individuals with mild cases, moderate cases, severe cases, and critical cases. A previous study has demonstrated that MXSG exhibits similar antiviral activity to oseltamivir and broad-spectrum inhibitory activity in mice infected with influenza A virus (Li et al., 2017; Zou et al., 2018). This activity has also been confirmed in the LHQW and JHQG treatment of H1N1 patients (Duan et al., 2011; Wang et al., 2011). Also, MXSG can downregulate chemokines, inhibit inflammation response, and ameliorate the ALI in model rats (Ma et al., 2014; Fei et al., 2019). However, the overall understanding of the therapeutic effect and potential mechanisms of MXSG in the COVID-19 remains elusive.

Here, we dissected the chemical components of MXSG by liquid chromatography–mass spectrometry (LC-ESI-MS/MS) and analyzed the intervention pathways of MXSG based on components detected through network pharmacology. At the same time, the therapeutic effect of MXSG on COVID-19 was explained through published articles, and the relevant regulatory mechanism was proposed. Then, in this study, the regulatory effect of MXSG on inflammatory lung injury was validated through transcriptome results. In summary, our study suggests that MXSG inhibits viral invasion, proliferation, and mitigation of virus-induced lung injury, which may be a key mechanism of its therapeutic effect on COVID-19. These results provide experience for the treatment of infectious diseases and lung injury.

## Results

### Characterization of Chemical Constituents in Ma Xing Shi Gan Decoction

**FIGURE 1:**
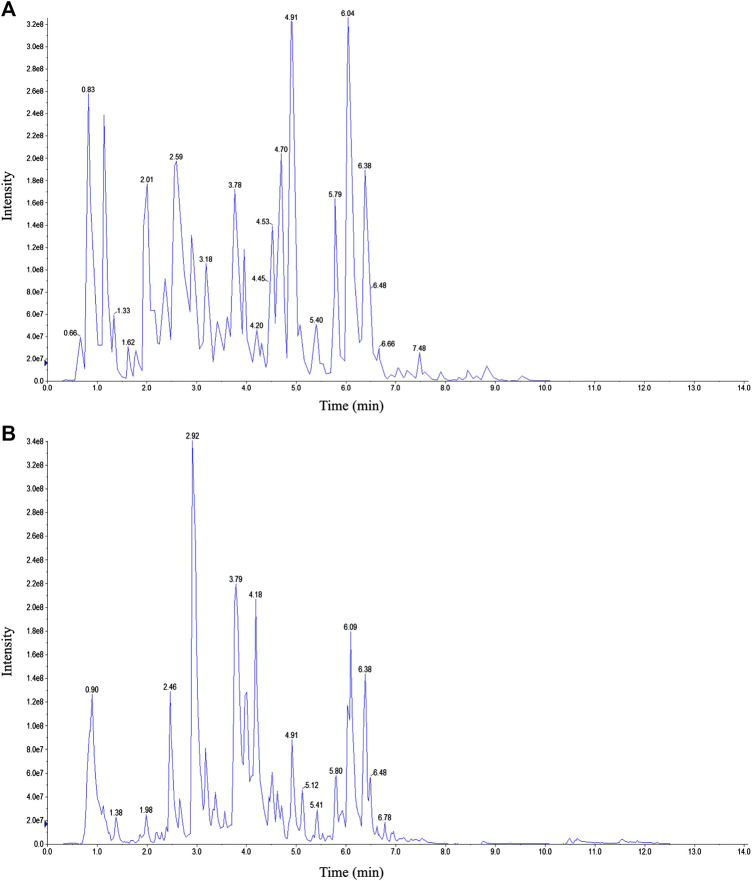
Representative total ion chromatogram MXSG. **(A)** Positive mode. **(B)** Negative mode.

Representative total ion chromatograms obtained by LC-ESI-MS/MS are shown in Figure 1. After peak integration, 126 peaks were detected as individual compounds. Based on the MetWare database and the published database of metabolite information, qualitative analysis was conducted on the primary and secondary spectrum data of mass spectrometry. As a result, 97 components were identified from MXSG. The compounds identified from MXSG were listed in Supplementary Table S1, including 40 flavonoids, 18 phenolic acids, 16 alkaloids, 10 terpenes, five lignans and coumarins, one quinone, and other types of compounds.

### Characterization of Potential Therapeutic Targets of Ma Xing Shi Gan Decoction

**FIGURE 2:**
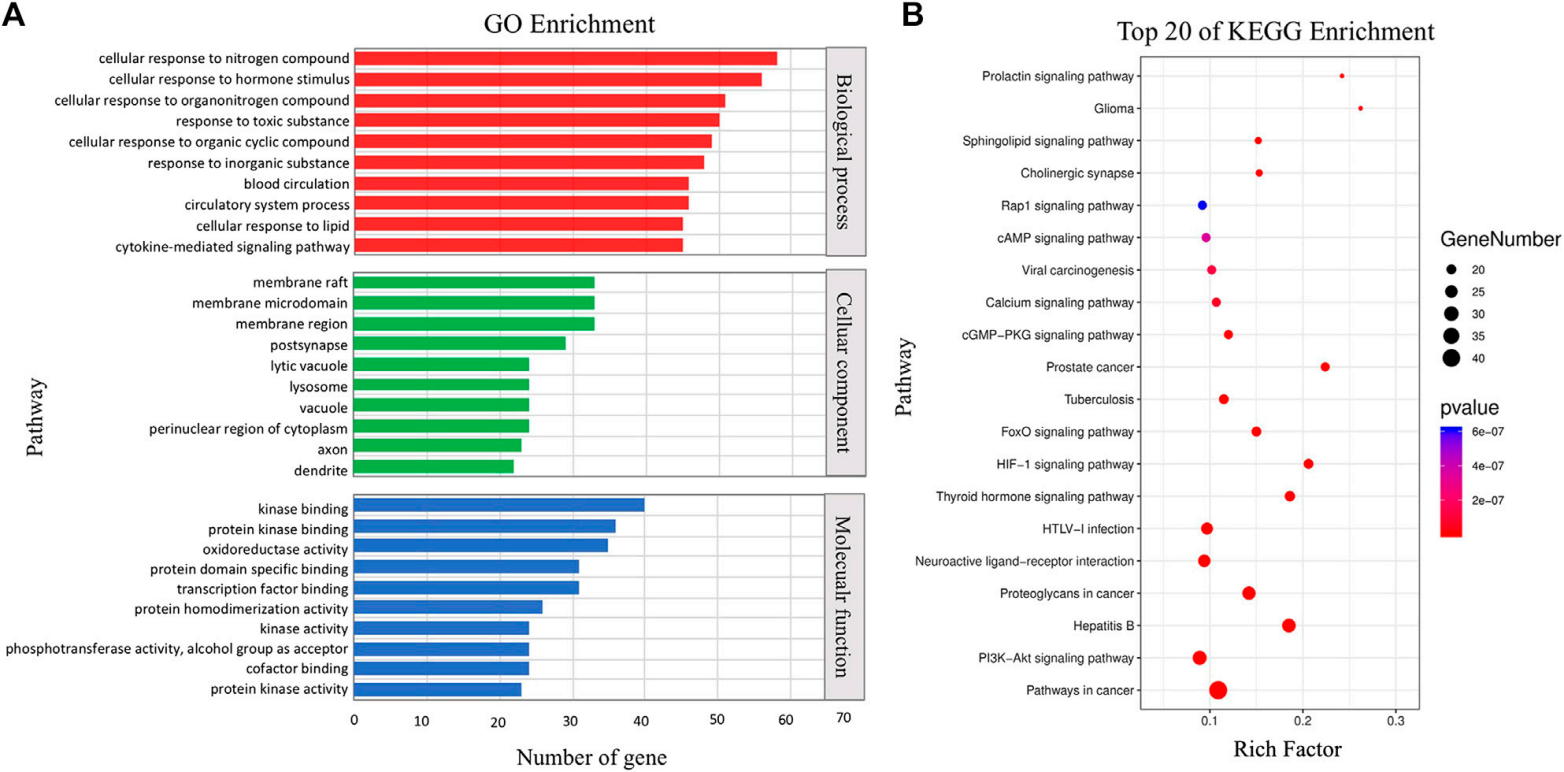
Functional analysis of MXSG. **(A)** GO enrichment of related genes. **(B)** Top 20 pathways enriched by the KEGG method.

The potential therapeutic target network of MXSG was presented in Figure 2. We first collected the targets of compounds identified by LC-ESI-MS/MS. Among the 97 compounds detected, 54 corresponding targets were obtained through database retrieval, and a total of 204 targets were obtained after merging and deleting. Then, GO and KEGG enrichment analysis was performed on the target information (Figure 2). In the GO enrichment analysis results, MXSG mainly played an intervention role by interfering with cellular processes and metabolic processes. The intervention mainly affects the cell membrane structure, and the main target molecules participate in the protein binding process and catalytic function. KEGG analysis showed that the role of MXSG was mainly to interfere with tumor-related pathways and viral infection–related pathways. The target information is in Supplementary Table S2.

### Efficacy of Ma Xing Shi Gan Decoction Against COVID-19 From Published Research Evaluation

MXSG is one of the most frequently used and valid prescriptions for COVID-19 prevention and control programs. It is the fundamental component of three drugs and three formulas*,* including LHQW, JHQG, QFPD, HSBD, and XFBD promulgated by China’s National Health Commission. We made a summary of MXSG and its extended formula formulation, effects, and clinical features (Supplementary Table S3).

And then, we made a summary of therapeutic effects of MXSG or its extended formula for SARS-CoV-2 or H1N1 infection. The results showed that MXSG was effective in treating influenza or viral pneumonia in both animal and clinical studies. The animal research involved three animal models of type A influenza virus infection, including BALB/c mice, KM mice, and WT mice. In the clinical studies, a total of 4,596 cases patients with SARS-CoV-2 or H1N1 infection were involved, including clinical observation studies, prospective cohort studies, retrospective studies, double-blinded randomized control trials, and randomized double-blind positive controlled clinical trial studies (Table 1).

**TABLE 1:** Efficacy evaluation of MXSG or its extended formula for influenza virus infection.

Drug	Therapeutic effects	Methodology	References
MXSG	Alleviated lung inflammatory, reduced lung weight index	Animal studies: MXSG treatment in type A influenza virus infection in BALB/c mice	Zhang et al. (2013)
	Alleviated colon tissue pathological injury induced by influenza virus lung infection	Animal studies: MXSG treatment in type A influenza virus infection in KM mice	Zou et al. (2018)
	Antiviral, improved lung inflammation and cytokines balance, protected the immune organ	Animal studies: MXSG treatment in type A influenza virus infection in WT mice	Li et al. (2017)
	Definite curative effect, no obvious adverse reaction	Clinical observation: 40 COVID-19 were treated with usual treatment combined with MXSG	Qu et al. (2020)
LHQW	Increased the symptom recovery rate and median time (fever, fatigue, and coughing), and improved the rate of chest CT manifestations and clinical cure	Prospective cohort study: 284 patients with COVID-19 were randomly divided into two groups (142 in each treatment group and control group), which received usual treatment alone or in combination with LHQW	Hu et al. (2020)
	Compared with oseltamivir, similar therapeutic effects were achieved, with shorter duration of disease and viral shedding, and reduced the severity of illness and the duration of symptoms	Randomized, double blind, positive controlled clinical trial: 244 patients with influenza A (H1N1) virus, were randomized to two treatment groups (112 cases in each group). Each group assigned to receive either LHQW or oseltamivir	Duan et al. (2011)
	Significantly improve the symptoms, no obvious adverse reaction	Retrospective study: 101 COVID-19 suspected case, 63 cases were received usual treatment and combination with LHQW, 38 cases were received usual treatment	Lv et al. (2020)
JHQG	Oseltamivir and JHQG, alone and in combination, reduced the duration of the fever	Prospective cohort study: 410 cases with confirmed H1N1, were randomly assigned receive oseltamivir/JHQG treatment alone or in combination (control 103 cases, oseltamivir 102 cases, JHQG 103 cases, and oseltamivir plus JHQG 2,013 cases)	Wang et al. (2011)
	The clinical symptoms of fever, cough, fatigue, and expectoration were reduce compared with control group; psychological anxiety of patients was relieved	Clinical observation: 123 COVID-19 patients were randomly divided (1:2) into routine treatment alone or combined with JHQG	Duan et al. (2010)
	Routinely low dose JHQG was effective and safe in treating patients with influenza	Double blinded randomized control trial: 136 influenza patients were randomized by stratification into three groups, high-dose JHQG group (44 cases), low-dose JHQG group (45 cases), and placebo control group (47 cases)	Li et al. (2013a)
	Definite curative effect, improve the clinical symptoms, reduce the deterioration of the disease, also has the effect on the immunological index	Clinical observation: 102 mild cases and moderate cases with Covid-19, were randomized to receive usual treatment alone or in combination with JHQG. Retrospective study: 80 COVID-19 patients were received routine treatment in combination with JHQG	National Health Commission of the People’s Republic of China (2020), Liu et al. (2020)
QFPD	Reduced the length of hospital stay, improved clinical symptoms, stopped the deterioration of the disease, reduced the death rate, and weakened the harm of the epidemic	Retrospective study: 60 COVID-19 patients were received usual treatment alone (30 cases) or in combination with QFPD (30 cases). Clinical observation: 1,263 cases with Covid-19, 57 severe cases with Covid-19, patients were received usual treatment in combination with QFPD	National Health Commission of the People’s Republic of China (2020), Li et al. (2020b)
XFBD	In mild and normal patients, improved clinical symptoms, controlled the progression of the disease, alleviated inflammatory, and improved the lymphocyte count	Clinical observation: 1,120 cases with Covid-19 (XFBD group 70 cases, control 50 cases). 240 mild cases and moderate cases with Covid-19. 3,500 mild and moderate cases with Covid-19. Patients were randomized to receive usual treatment alone or in combination with XFBD	National Health Commission of the People’s Republic of China (2020)
HSBD	The effectiveness and safety were determined, improved pulmonary inflammation and clinical symptoms, and shortened duration of viral shedding and hospital stay. No drug-related adverse reactions were found	Clinical observation: 175 severe cases with Covid-19. 2,124 moderate cases with Covid-19. 3,894 mild cases and moderate cases with Covid-19 (HSBD group 452 cases). Patients were randomly divided into single routine treatment or combined HSBD	National Health Commission of the People’s Republic of China (2020)

MXSG has been showing the effects of antiviral (reduction of the duration of viral shedding), ameliorated the clinical symptoms (fever, fatigue, coughing, etc.), inhibited progression (reducing the deterioration of the disease), regulated immune inflammation (alleviating inflammatory, improving the lymphocyte count, inhibiting the release of inflammatory cytokine, etc.), and depressed lung injury (pathological scores, alveolar-capillary barrier damage, pulmonary edema, and inflammatory factors were reduced.) in basic and clinical studies. In particular, the effect of MXSG for improving viral lung injury was close to oseltamivir. Moreover, in these studies, no noticeable drug-related adverse reactions were found between MXSG or its extended formula (Table 1).

### Molecular Targets and Mechanism From Published Biological Evaluation

#### Ma Xing Shi Gan Decoction Inhibits the Adsorption and Replication of Virus

MXSG might directly inhibit the adsorption and replication of SARS-CoV-2 at the viral entry step. LMEP, LEP, DPEP, and (+)-catechin are active ingredients of *Ephedra sinica* Stapf*.* Glycyrrhizin is an active ingredient of *Glycyrrhiza glabra* L. We summarized the action of MXSG or its active components on influenza and coronavirus to validate our hypothesis (Table 2). MXSG or its active ingredients could inhibit both viral adsorption and penetration by inducing disruption of the viral particle or affecting the interacts with the cell membrane. Moreover, they also have a potent inhibitory effect on virus replication.

**TABLE 2:** Experimental evidence of MXSG or its herbal/active ingredients for anti-influenza virus.

MXSG/ingredient	Target	Mechanism	Methodology	References
MXSG	AKT phosphorylation↓, PI3K↓	Inhibited both viral adsorption and penetration; induced disruption of the viral particle	MXSG against influenza virus A/WSN/33 in MDCK cells	Hsieh et al. (2012)
	Neuraminidase↓	Prevented the proliferation of influenza virus	MXSG against type A influenza virus infection in BALB/c mice	Zhang et al. (2013)
MH	Acidification of endosomes and lysosomes↓	Inhibited virus growth	MH against influenza A/PR/8 virus in MDCK cells	Mantani et al. (1999)
LMEP, LEP and DPEP	NA	Inhibited the proliferation	MXSG treatment in influenza A in MDCK cells and male ICR mice	Wei et al. (2019)
(+)-Catechin	Acidification of endosomes and lysosomes↓	Inhibited virus growth	(+)-Catechin treatment in influenza A/PR/8 virus in MDCK cells	Mantani et al. (2001)
Glycyrrhizin	NA	Lower membrane fluidity and inhibited virus entry	Glycyrrhizin treatment in influenza A/Aichi/2/68 virus in MDCK cells	Harada (2005)
	NA	Reduced cell membrane endocytotic activity and reduced virus uptake	Glycyrrhizin treatment in influenza A virus (IAV) in MDCK/A549/Hfl-1	Wolkerstorfer et al. (2009)
	NA	Inhibited virus proliferation, adsorption and penetration	Glycyrrhizin treatment in SARS-associated coronavirus in Vero cell culture (FFM-1 and FFM-2)	Cinatl et al. (2003)

MH, *Ephedra sinica* Stapf; GC, *Glycyrrhiza glabra* L.; LMEP, L-methylephedrin; LEP, L-ephedrine; DPEP, D-pseudo-ephedrine; MDCK, Madin-Darby canine kidney; A549, human endothelial lung cells; Hfl-1, human lung fibroblast cells.

#### Ma Xing Shi Gan Decoction Inhibits the Inflammatory Response Through Toll-Like Receptor Signaling Pathway

TLRs are at the interface of innate immune activation in an infected environment by responding to a variety of microorganisms and endogenous ligands (Mollen et al., 2006). MXSG could target TLRs and the inflammatory response triggered by TLRs. Resulting in multiple phenotype changes, such as inhibiting the release of inflammatory cytokines, reduces lung inflammation. Experimental evidence that MXSG or its active ingredients for inhibiting inflammatory lung injury were summarized (Table 3).

**TABLE 3:** MXSG or its active ingredients for inhibiting the inflammatory lung injury.

MXSG/ingredient	Target		Mechanism	Methodology	References
MXSG	TNF-α, IL-1β and IL-6↓, TLR4, MyD88, and TRAF6↓	Inhibited TLR4-MyD88-TRAF6 signaling pathway and release of inflammatory cytokines, alleviated the inflammation reaction		MXSG against type A influenza virus infection in WT mice	Li et al. (2017)
	MCP-1↓	Inhibited inflammation reaction		MXSG treatment in type A influenza virus infection in KM mice	Zou et al. (2018)
	TNF-α, IL-1β and IL-6↓, ICAM-1, TLR4, cav-1, Src and NF-κB↓, claudin-5, JAM-1 and occludin↑, p-cav-1↓, and MPO↓	Inhibited the release of inflammatory cytokines and alleviated the inflammation reaction		MXSG posttreatment in LPS-induced male Sprague–Dawley rats ALI	Ma et al. (2014)
	TNF-α, IL-1β and IL-6↓, MPO↓, HMGB1, TLR4, MyD88, and p-p65↓	Inhibited HMGB1/TLR4/NF-κB signaling and release of inflammatory cytokines, and alleviated the inflammation reaction		MXSG treatment in PM2.5 induced male Sprague-Dawley rats ALI	Fei et al. (2019)
Glycyrrhizin	F12, F13b, F9, and AT3	These proteins were involved in the conversion of zymogen to serine protease, affecting the regulation of innate immunity		MXSG treatment in LPS-induced rats ALI	Yang et al. (2020)
	NA	Stimulation of IFN-gamma production by T cells		GL treatment in mice infected with influenza virus A2	Utsunomiya et al. (1997)
	TNF-α, IL-1β, and IL-6↓, TLR4, COX-2, MPO, iNOS, and NF-κB↓	Inhibition of the TLR-4/NF-κB signaling pathway		GL treatment in LPS-induced BALB/c mice ALI	Lee et al. (2019)
	Tlr2↑, MIP-2, KC, IL-4, IL-6, GM-CSF, NF-κB, and IFN-γ↓	Inhibition of the TLR signaling pathway		GL treatment in LPS-induced BALB/c nude mice ALI	Kong et al. (2019)
	TLR2, MyD88, and NF-κB↓	Downregulate TLR2 signaling inhibit I/R-induced inflammatory response		GL could ameliorate I/R induced male BALB/C mice lung injury	Fei et al. (2017)
Licochalcone A	TNF-α, IL-1β, and IL-6↓	Anti-inflammation reaction and alleviated inflammatory lung injury		Lico A treatment in LPS-induced male BALB/c mice ALI	Chu et al. (2012)
LEP, DPEP	IL-1β, TNF-α, TLR3, TLR4, TLR7, MyD88, NF-κB p65, and RIG-1, IFN-γ, and IL-10↓	Adjusting the TLRs and RIG-1 pathways alleviating lung injury		LEP, DPEP treatment in influenza A in male ICR mice	Wei et al. (2019)

GL, glycyrrhizin; LMEP, L-methylephedrin; LEP, L-ephedrine; DPEP, D-pseudo-ephedrine; Lico A, licochalcone A; LPS, lipopolysaccharide; ALI, acute lung injury; TLRs, toll-like receptors; I/R, ischemia–reperfusion.

### Validation From *In Vivo* Transcriptome of Lipopolysaccharide-Induced Lung Injury

#### Differential Expression Analysis

**FIGURE 3:**
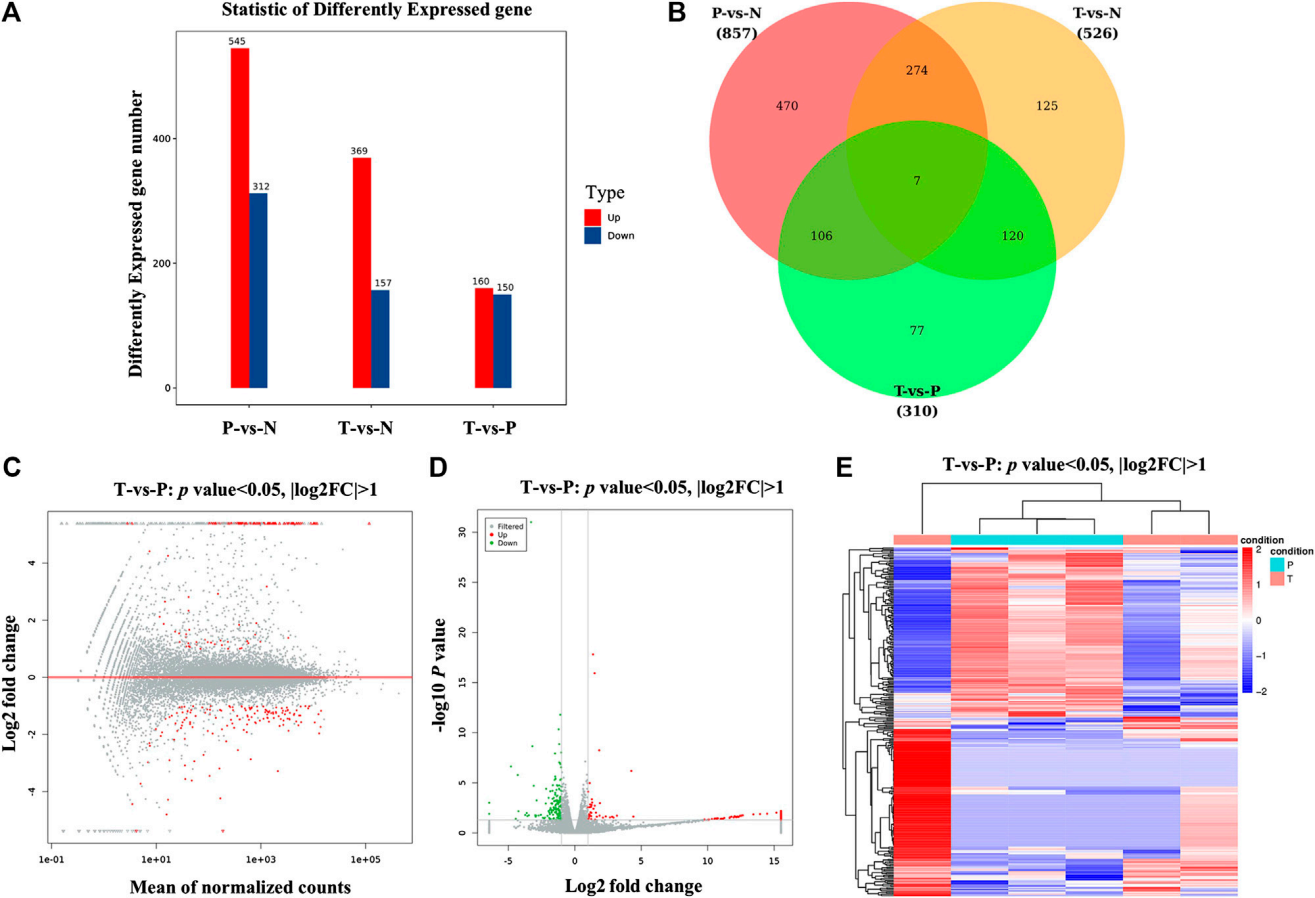
Screening and hierarchical clustering analysis of DEGs. **(A)** Histogram of DEGs in groups. The horizontal axis is the comparison groups. The vertical axis is the number of DEGs in the comparison group, in which Up is the number of significantly upregulated genes and Down is the number of significantly downregulated genes. **(B)** Venn plots of DEGs in groups. **(C)** MA plots of DEGs, the horizontal axis indicated standardized expression mean in all samples, and vertical axis indicated log2 fold change. Red dots indicated significant DEGs. **(D)** Volcano plot of DEGs between MXSG group and pneumonia group. Red and green dots indicated significantly upregulated and downregulated genes. Gray dots indicated nonsignificant DEGs. The horizontal axis indicated where log2 fold change, and vertical axis indicated where −log10 *p* value. **(E)** Clustering analysis of DEGs between MXSG group and pneumonia group. MXSG group and pneumonia group were shown as red and cyan along the horizontal bar, and genes were shown along the vertical bar. Upregulated genes were shown as red and downregulated genes were shown as green. N, normal group; P, pneumonia model group; T, MXSG group.

Differential expression analysis revealed a total of 310 differential genes after treatment with MXSG. There were 160 upregulated genes and 150 downregulated genes (*p* value < 0.05, |log2FC| > 1; Figures 3A,B). According to the MA and volcano plot of differentially expressed genes (DEGs) between the MXSG group and the pneumonia group, the upregulated and downregulated DEGs showed significant differences (Figures 3C,D). The cluster analysis of the DEG level also showed that the biological function significantly changes after the intervention of MXSG (Figure 3E).

#### Differentially Expressed Genes’ KEGG Pathway Enrichment Analysis

**FIGURE 4:**
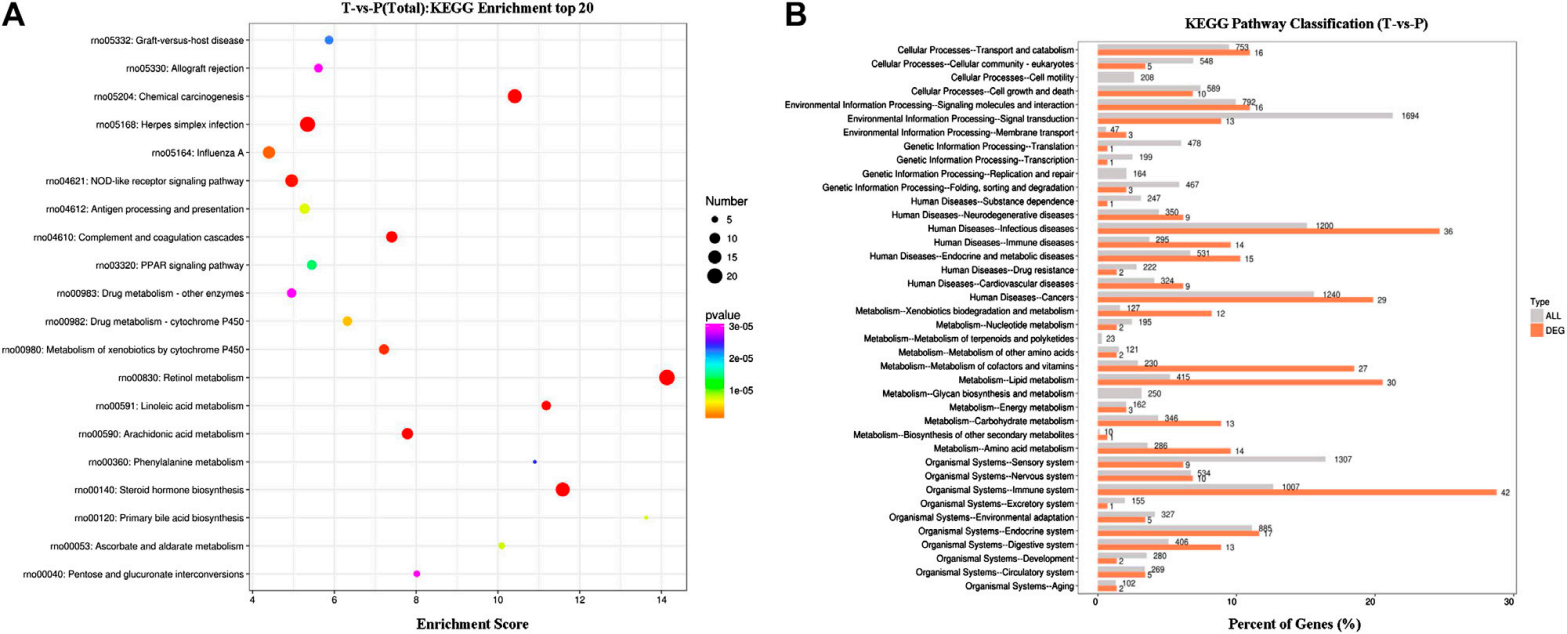
KEGG enrichment results. **(A)** Bubble map of the top20 KEGG pathways. **(B)** Comparison of histogram of KEGG level 2 distribution between DEGs and all gene. The horizontal axis is the ratio (%) of the genes annotated to each level 2 metabolic pathway and the total number of all genes annotated to the KEGG pathway. The vertical axis represents the name of level 2 pathway, and the number to the right of the column represents the number of DEG notes under the level 2 pathway.

DEGs’ KEGG pathway enrichment analysis indicated that 20 pathways of the transcription genes in lung tissues were enriched after the intervention of MXSG, including retinol metabolism, steroid hormone biosynthesis, complement and coagulation cascades, chemical carcinogenesis, herpes simplex infection, arachidonic acid metabolism, linoleic acid metabolism, NOD-like receptor signaling pathway, metabolism of xenobiotics by cytochrome P450, influenza A, drug metabolism–cytochrome P450, antigen processing and presentation, primary bile acid biosynthesis, ascorbate and alternate metabolism, PPAR signaling pathway, graft versus host disease, phenylalanine metabolism, pentose and glucuronate interconversions, drug metabolism—other enzymes, and allograft rejection (Figure 4). According to the KEGG secondary classification, the genes with more differences were correlated with the endocrine system, immune system, lipid metabolism, metabolism of cofactors and vitamins, and infectious diseases (Figure 4).

#### The Enrichment of Differentially expressed genes in Phosphatidylinositide 3-Kinases/Protein Kinase and Toll-Like Receptor Signaling Pathways

According to the KEGG enrichment pathway, the upregulated genes in the PI3K/Akt signaling pathway include REDD1, ECM, and CREB, downregulated genes. In the toll-like receptor signaling pathway, downregulated genes include IRF7, STAT1, and IP-10, and treatment with MXSG (Supplementary Figure S1).

## Discussion

**FIGURE 5:**
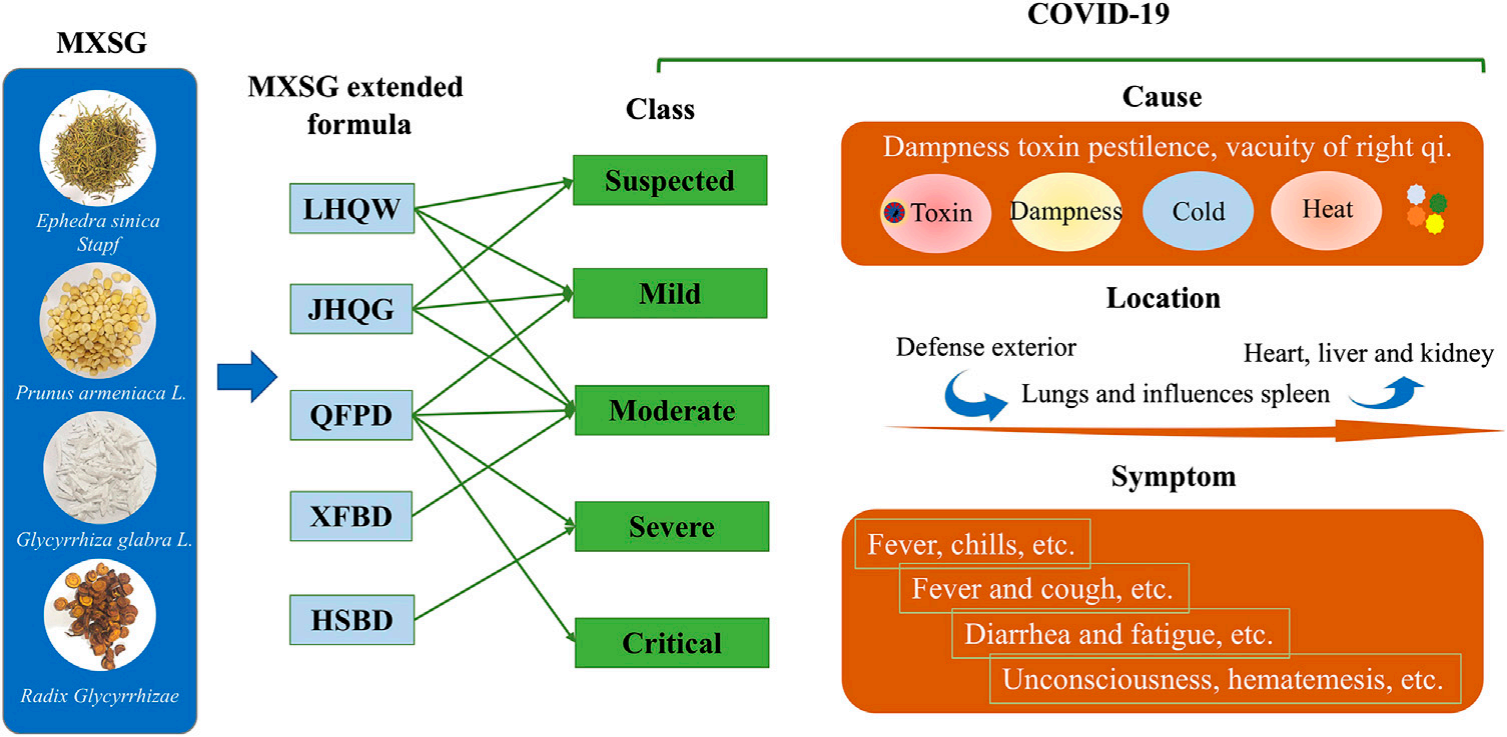
TCM theory of MXSG in the treatment of COVID-19. Information is from the latest guideline for the diagnosis and treatment of COVID-19 issued by the National Health Commission of the People’s Republic and the published literatures (Fan et al., 2020; Luo et al., 2020; Tong et al., 2020). The green arrows indicated this drug is recommended for the corresponding phase of COVID-19 treatment in the latest guideline for the diagnosis and treatment of COVID-19.

TCM theory of MXSG in the treatment of COVID-19. Chinese medical specialist confirms that COVID-19 belongs to the category of epidemic disease in TCM. Dampness toxin pestilence and vacuity of right qi are the main cause. The toxin combining with the dampness pathogen is the main TCM pathogenesis of COVID-19. It also includes cold pathogens and hot pathogens. The pathological evolution of SARS-CoV-2 in TCM can be summarized as pathogenic factors invading defense exterior in early stage, and then influences the lungs and spleen function, finally involves heart, liver and kidney. (Gu et al., 2020; Luo et al., 2020). It will cause some typical lung symptoms, including fever and cough. Besides, a few people also develop symptoms of the spleen, such as diarrhea and fatigue (Fan et al., 2020; Tong et al., 2020) (Figure 5). MXSG has a history of 1,800 years, from Treatise on Cold Damage. It is the core prescription of TCM to treat cough and asthma, and has the functions of clearing heat and preventing asthma, dispersing lung, and relieving cough (Supplementary Table S3). In this decoction, *Ephedra sinica* Stapf is a warm and dissipating acridity drug, adept in dispersing lung qi, opening the interstice structures, effusing wind cold. *Gypsum fibrosum* (calcium sulfate) has a medicinal property to treat cold, good at clearing lung fire, releasing flesh, and abating heat. *Prunus armeniaca* L*.*, as a descending qi with the bitter-warm drug, has the effect of cough-suppressing phlegm transforming. *Glycyrrhiza glabra* L. (licorice) is a harmonizing drug, relaxing the middle and supplementing vacuity, and clearing heat and detoxification. Studies show that MXSG is applied as a basic prescription in the treatment of SARS, H1N1, or MERS, and has achieved satisfactory efficacy (Wang et al., 2011; Li et al., 2013b; Zhang et al., 2013; Zou et al., 2018). In the clinical prevention and treatment of COVID-19 (Li et al., 2020a; Tang et al., 2020), MXSG has the highest frequency in China’s the national and provincial prevention and treatment programs and applied to COVID-19 patients’ both suspected cases, and confirmed individuals with mild cases, moderate cases, severe cases, and critical cases, and has become the core prescription of pulmonary infection.

The efficacy of MXSG in the treatment of COVID-19. Clinical manifestations of COVID-19 include fever, cough, fatigue, myalgia, diarrhea, normal or decreased white blood cell counts, and radiological evidence of pneumonia (Henry and Vikse, 2020; Li and De Clercq, 2020; Stower, 2020; Xiong et al., 2020). Severe patients usually develop dyspnea and/or hypoxemia 1 week after onset. In severe cases, acute respiratory distress syndrome, sepsis, refractory metabolic acidosis, coagulation disorders, and multi-organ failure may develop rapidly. These symptoms are similar to those of SARS-COV, H1N1, and MERs-CoV infections. MXSG and the extended formula have been showing the effects of antiviral, ameliorated the clinical symptoms, inhibited progression, regulated immune inflammation, and depressed lung injury in basic and clinical studies (Table 1). In particular, the effect of MXSG for improving viral lung injury was close to that of oseltamivir in animal studies (Zhang et al., 2013; Li et al., 2017; Zou et al., 2018). And similarly, JHQG alone and in combination, oseltamivir reduced time to fever resolution in patients with influenza A (H1N1) virus (Wang et al., 2011). Compared with oseltamivir, LHQW also achieved a similar therapeutic effectiveness reduction of the duration of symptoms and viral shedding, and reduced the severity of illness in patients with influenza A (H1N1) virus (Duan et al., 2011). Moreover, in these studies, no noticeable drug-related adverse reactions were found between MXSG and its extension. These show that MXSG is a relatively safe and effective treatment for influenza and viral pneumonia.

The mechanism of MXSG in the treatment of COVID-19. Besides chemical methods and literature surveys, network pharmacology is also an effective way to decipher the effective components and comprehensive information of Chinese medicine (Jiang et al., 2020). Although the web-based pharmacology strategy has the limitations of database itself defects and the uncertainty of active ingredient function prediction (Huang et al., 2020), the strategy will facilitate the mechanistic investigations of these clinically effective TCMs on COVID-19 to some extent (Jiang et al., 2020).

In this study, we analyzed the chemical composition of MXSG using LC-ESI-MS/MS and carried out GO and KEGG enrichment analysis on the targets of its composition. The results showed that MXSG mainly interfered with cellular and metabolic processes. The intervention mainly affects the cell membrane structure, and the main target molecules are involved in the protein binding process and catalytic function. KEGG analysis showed that the central role of MXSG was to interfere with the viral infection–related pathway and the PI3K/AKT signaling pathway (Figure 2). In the result of summarization, MXSG and its ingredients can inhibit influenza/coronavirus virus replication and invasion (Table 2). Hsieh et al. (2012) reported that MXSG could inhibit the synthesis of both viral RNA and protein, disrupt viral surface structure, and block the virus entry phase. More interestingly, in this study, it was demonstrated that virus intrusion is regulated by the PI3K/AKT signaling pathway, which was inhibited by MXSG. In addition, MH, GC, LMEP, LEP, and DPEP have been reported to prevent virus entry or proliferation (Mantani et al., 1999; Mantani et al., 2001; Cinatl et al., 2003). Thus, MXSG might directly inhibit the adsorption and replication of SARS-CoV-2 at the viral entry step.

**FIGURE 6:**
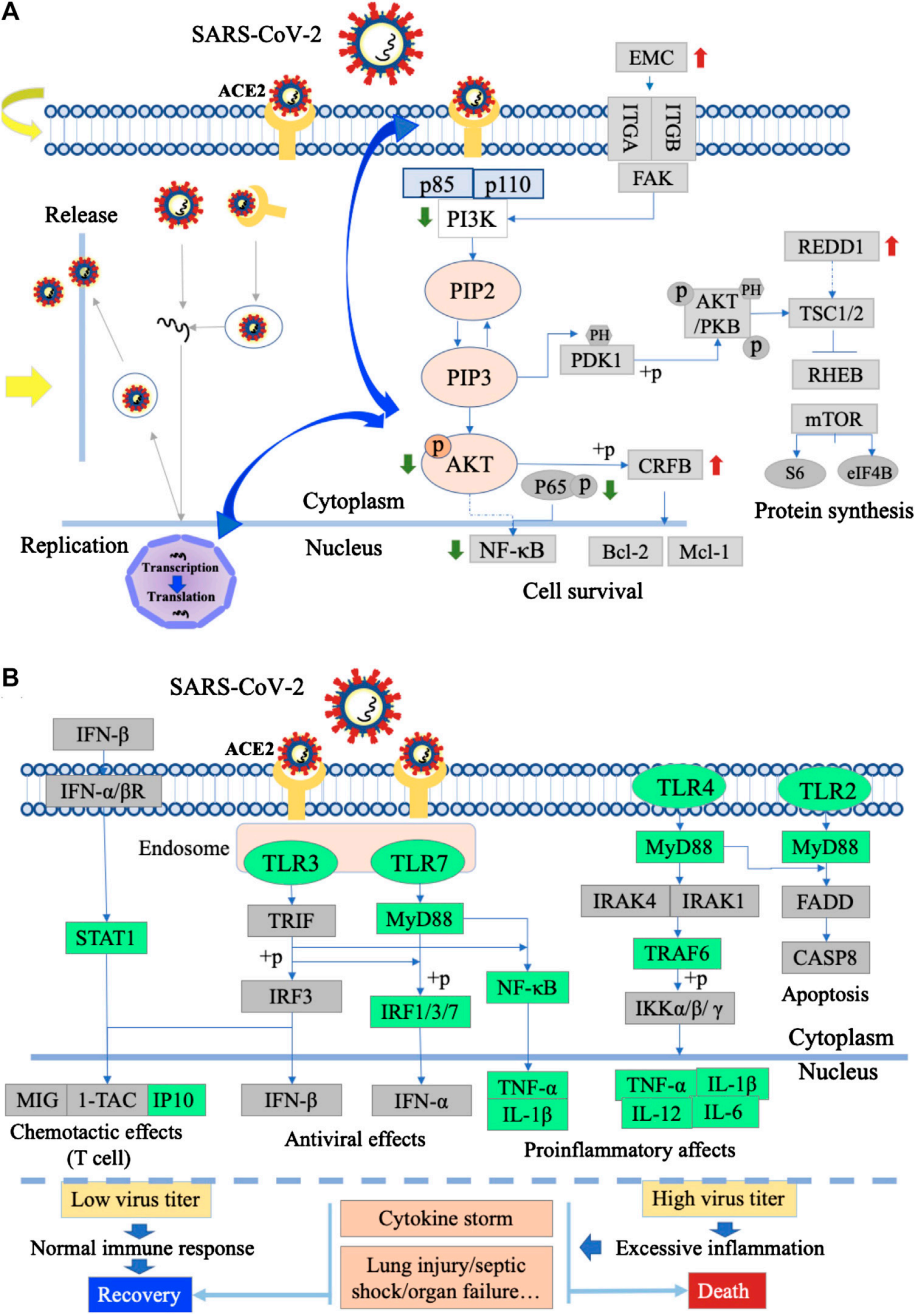
Action mechanism diagram of MXSG. **(A)** SARS-CoV-2 entry, replication, and PI3K/AKT signaling pathway infected with SARS-CoV-2. The green arrows indicated downregulated genes, and the red arrows indicated upregulated genes with MXSG or its ingredients intervention. The yellow arrows indicated MXSG regulates the intrusion and replication of viruses. The blue arrows indicated MXSG might play antiviral effect in regulating the interaction between PI3K/AKT and virus invasion and replication. **(B)** Toll-like receptors signaling pathway infected with the severe/mild SARS-CoV-2. The green box indicated downregulated genes with MXSG or its ingredient intervention.

MXSG can effectively alleviate inflammatory lung injury. The early immune inflammatory response is essential for virus clearance. Pattern recognition receptors recognize the pathogen-associated molecular patterns. The initiation of the inflammatory response depends on the recognition of pattern recognition receptors. TLRs have been reported, which enables the recognition of influenza viruses by pattern recognition receptors. It can have both protective and detrimental effects during infection. Innate responses are armaments that the host can use to prevent or slow viral replication early in infection (Krammer et al., 2018) and, however, are the cause of severe conditions such as lung damage caused by excessive inflammation (Short et al., 2014; Biondo et al., 2019) (Figure 6B). Various studies revealed that MXSG and its ingredients could inhibit inflammation reaction by intervening TLR signaling (Table 3). This effect effectively reduced the level of inflammatory cytokines and improved lung injury (Chu et al., 2012; Ma et al., 2014; Fei et al., 2017; Li et al., 2017; Wei et al., 2019). In this study, the regulatory effect of MXSG on inflammatory lung injury was validated through transcriptome results. After MXSG intervention, several genes on the toll-like receptor signaling pathway were found to be significantly altered. Thus, MXSG may inhibit SARS-CoV-2 inflammatory lung injury by regulating the TLR signaling pathway. However, a more precise regulatory mechanism still needs to be demonstrated in future studies.

This study incorporates chemical methods, literature surveys, and network pharmacology ways to decipher the effective components and comprehensive information of MXSG. Through this study, the active components of MXSG were analyzed, the curative effect of MXSG on COVID-19 was proved, and the possible mechanism of MXSG in the treatment of COVID-19 was proposed. Then, transcriptome experiments were used to preliminarily verify the possible mechanism. Undeniably, there are some limitations in this study, including that the proposed mechanism of MXSG has not been effectively verified, and the selection of animal models cannot be fully recognized. Therefore, our research group will carry out further research in the future.

## Conclusion

In this study, we analyzed the main active components of MXSG and predicted its intervention mechanism. Through literature analysis, it is preliminarily determined that MXSG is an essential prescription for the treatment of COVID-19. It has the curative effect of antivirus, improving clinical symptoms, regulating immune inflammation, and inhibiting lung injury. Further, we found that MXSG might directly inhibit the adsorption and replication of SARS-CoV-2 at the viral entry step. In addition, it may play an anti-inflammatory and immune regulatory role to prevent cytokine storm, relieving lung injury through TLR signaling pathway. However, the specific mechanism of MXSG in the treatment of COVID-19 still needs further research.

## Materials and Methods

### Component Detection, Target Prediction, and Functional Analysis of Ma Xing Shi Gan Decoction

The Pharmacy Department provided all crude drugs of Ma Xing Shi Gan Decoction, Dongfang Hospital Affiliated to Beijing University of Chinese Medicine (Beijing, China). They were purchased from the Beijing Tcmages Pharmaceutical Co., Ltd., (Shunyi district, Beijing, Datong Road). *Ephedra sinica* Stapf [Ephedraceae] (Ma Huang) were collected from the province of Henan, China; *Prunus armeniaca* L. [Rosaceae] (Ku Xing Ren) from the province of Hebei, China; *Glycyrrhiza glabra* L. [Fabaceae] (Gan Cao; licorice) from the province of Ningxia, China; and *Gypsum fibrosum* (Shi Gao; calcium sulfate) from the province of Shanxi, China. The quality of crude drugs was strictly performed according to Good Manufacturing Practice for Drugs to guarantee quality control (Chinese FDA). Furthermore, these species were authenticated by Prof. Xiaohong Gu (Beijing University of Chinese Medicine) before use. Voucher specimens (no. BUCM-LI-2019001 for *Ephedrae herba*; no. BUCM-LI-2019002 for *Armeniacae amarum semen*; no. BUCM-LI-2019003 for *Glycyrrhizae radix preparata*; no. BUCM-LI-2019004 for *Gypsum fibrosum*) were deposited in the School of Traditional Chinese medicine, Beijing University of Chinese Medicine (Beijing, China). The Pharmacy Department provided the test samples of Ma Xing Shi Gan Decoction according to the prescription proportion of “Treatise on Cold Damage” (*Ephedra sinica* Stapf:*Prunus armeniaca* L.:*Glycyrrhiza glabra* L*.*:*Gypsum fibrosum* = 2:1.5:1:4), Dongfang Hospital Affiliated to Beijing University of Chinese Medicine (Beijing, China). A method of decoction was used to extract the herb, and then the extracts were concentrated and dried to form granules (Dongfang Hospital Affiliated to Beijing University of Chinese Medicine). The characteristics of each component in MXSG, based on traditional prescription theory, were shown in Supplementary Table S4.

The reagents methanol, ethanol, and acetonitrile of HPLC grade were provided by Merck Chemicals (Darmstadt, Germany). Standard (DMSO) of HPLC grade was provided from BioBioPha (http://www.biobiopha.com/) and Sigma-Aldrich (St. Louis, MO, United States). A Milli-Q system (Millipore Corp, Millipore, MA, United States) was used to provide ultrapure water.

The freeze-dried MXSG was crushed using a mixer mill (MM 400, Retsch) with a zirconia bead for 1.5 min at 30 Hz. 100 mg powder was weighted and extracted overnight at 4°C with 1.0 ml 70% aqueous methanol. Following centrifugation at 10,000g for 10 min, the extracts were absorbed (CNWBOND Carbon-GCB SPE Cartridge, 250 mg, 3 ml; ANPEL, Shanghai, China, www.anpel.com.cn/cnw) and filtrated (SCAA-104, 0.22 μm pore size; ANPEL, Shanghai, China, http://www.anpel.com.cn/) before LC-ESI-MS/MS analysis.

The sample extracts were analyzed using an LC-ESI-MS/MS system (HPLC, Shim-pack UFLC SHIMADZU CBM30A system, www.shimadzu.com.cn/; MS, Applied Biosystems 6500 Q TRAP, www.appliedbiosystems.com.cn/). The analytical conditions were as follows, HPLC: column, Waters ACQUITY UPLC HSS T3 C18 (1.8 µm, 2.1 mm × 100 mm); solvent system, water (0.04% acetic acid): acetonitrile (0.04% acetic acid); gradient program, 100:0V/V at 0 min, 5:95V/V at 11.0 min, 5:95V/V at 12.0 min, 95:5V/V at 12.1 min, 95:5V/V at 15.0 min; flow rate, 0.40 ml/min; temperature, 40°C; injection volume: 2 μL. The effluent was alternatively connected to an ESI-triple quadrupole-linear ion trap (Q TRAP)-MS.

LIT and triple quadrupole (QQQ) scans were acquired on a triple quadrupole-linear ion trap mass spectrometer (Q TRAP), API 6500 Q TRAP LC/MS/MS system, equipped with an ESI Turbo Ion-Spray interface, operating in a positive ion mode and controlled by Analyst 1.6.3 software (AB Sciex). The ESI source operation parameters were as follows: an ion source, turbo spray; source temperature 500°C; ion spray voltage (IS) 5,500 V; ion source gas I (GSI), gas II (GSII), curtain gas (CUR) was set at 55, 60, and 25.0 psi, respectively; the collision gas (CAD) was high. Instrument tuning and mass calibration were performed with 10 and 100 μmol/L polypropylene glycol solutions in QQQ and LIT modes, respectively. QQQ scans were acquired as MRM experiments with collision gas (nitrogen) set to 5 psi. DP and CE for individual MRM transitions were done with further DP and CE optimization. A specific set of MRM transitions were monitored for each period according to the metabolites eluted within this period.

Based on the self-established MetWare database and the common database of metabolite information, qualitative analysis was conducted on the primary and secondary spectrum data of mass spectrometry. In the qualitative analysis of some substances, isotopic signals, repeated signals containing K^+^ ions, Na^+^ ions, and NH_4_^+^ ions, as well as repeated signals of fragments of other substances with larger molecular weight, are removed. Metabolite structure analytical reference MassBank (http://www.massbank.jp/), KNAPSAcK (http://kanaya.naist.jp/KNApSAcK/), HMDB (http://www.hmdb.ca/), MoTo DB (http://www.ab.wur.nl/moto/), and METLIN (http://metlin.scripps.edu/index.php), and other existing mass spectrometry public database.

Retrieval of the compounds detected targets from the Symmap database (http://www.symmap.org) and TCMSP database (http://lsp.nwsuaf.edu.cn/tcmsp.php). Then, target genes were uploaded to the Metascape platform (http://metascape.org/gp/index.html) for GO analysis and then uploaded to the DAVID platform (https://david.ncifcrf.gov/) for KEGG analysis. The results are shown by bar and bubble diagrams.

### Therapeutic Effects of Ma Xing Shi Gan Decoction

Information of MXSG or its extended formula is from corresponding drug labels and the latest guideline for the diagnosis and treatment of COVID-19 issued by the National Health Commission of the People’s Republic. Therapeutic effects of MXSG or its extended formula, and potential action mechanisms of MXSG and its ingredients for COVID-19 were summarized from published evaluation in PubMed (https://pubmed.ncbi.nlm.nih.gov/). Since some of the research was published only in Chinese, we also added a few published evaluations from CNKI (https://www.cnki.net). Since the symptoms of COVID-19 and H1N1 are highly similar, the therapeutic effects of MXSG or its extended formula were summarized to include the treatment of H1N1.

### *In Vivo* Transcriptome Experiment of Ma Xing Shi Gan Decoction

Eight- or nine-week-old male SD rats (110 g ± 10 g) were purchased from SPF (Beijing) Biotechnology Company. Experimental animals were maintained under specific pathogen-free conditions according to agency guidelines. The rats were kept with a 12-h light/dark cycle and with access to water and food *ad libitum*. The experimental procedures were approved by the Ethical Committee on Animal Research at the Beijing University of Chinese Medicine (BUCM-4-2019082701-3040) and conducted following the Guide for the Care and Use of Laboratory Animals established by the US National Institutes of Health. After 3 days of adaptive breeding, 30 rats were randomly divided into three groups (*n* = 10): normal group, pneumonia model group, and MXSG group. Next, the pneumonia model group and MXSG group were received, given 0.5 mg/ml LPS nebulization intervention, 30 min per day for three consecutive days. After 3 days, the MXSG group were intragastrically administered of MXSG once a day for 3 consecutive days (according to clinical guidelines, all doses were converted according to the equivalent dose of 0.018 for human and rat). The model group and the control group received an equal volume of saline accordingly. Rats were sacrificed after drug treatment on the condition of free drinking but without food for 12 h, then anesthetized with 10% chloral hydrate. Lung was collected for quick freezing. Furthermore, the samples were stored at −80°C refrigerator.

RNeasy Mini-Kit (QIAGEN, Valencia, CA) was used to simultaneously extract total RNA from the lungs of three groups of rats (normal, model, and MXSG). Later, DNA was digested using DNase and enriched with Oligo (dT) magnetic beads. Then, the destruction reagent was added to decompose the mRNA into short fragments, using the destroyed mRNA as a template and randomly using six bases. Primers synthesized single-stranded cDNA, and then the double-stranded reaction system was prepared to synthesize double-stranded cDNA and purify double-stranded cDNA. The purified double-stranded cDNA was terminal repaired, a tail was added, attached to the sequencing adapter, and finally, PCR amplification was performed. After the library was detected by Agilent 2100 Bioanalyzer, Illumina HiSeq™ two sequencer was used to sequence the library, and 125 bp or 150 bp double-ended data were generated. After quality inspection, Illumina sequencer was used for sequencing, and bioinformatics analysis was conducted according to the sequencing results.

DESeq software was used to standardize the counts of each sample gene (the basement value is used to estimate the expression quantity) and calculate the multiple difference. NB (negative binomial distribution test) was used to test the different significance of read numbers. Finally, significantly, DEGs were screened according to the difference in multiple and different significance test results. *p* value < 0.05 and fold change > 2 or fold change < 0.5 was set as the selection condition. Pathway analysis of differential expression was performed using the KEGG database (combined with KEGG annotation results), and the hypergeometric distribution test was used to calculate the significance of enrichment of DEGs in each pathway entry. The formula for calculating *p* value and enrichment fraction by hypergeometric distribution test is as follows:

$$\begin{array}{l} P=1-\overset{m-1}{\underset{i=1}{\sum }}\frac{\left(\begin{array}{c} M\\ i \end{array}\right)\left(\begin{array}{c} N-M\\ n-i \end{array}\right)}{\left(\begin{array}{c} N\\ n \end{array}\right)}\;.\\ \text{Enrichment}\;\text{score}=\frac{m/n}{M/N}, \end{array}$$

where *N* is the number of KEGG annotated genes in all genes, *n* is the number of genes with KEGG annotation in DEGs in *N*, *M* is the number of genes annotated with specific KEGG pathways in all genes, and *m* is the number of genes differentially expressed by specific KEGG pathways.

## Data Availability Statement

The RNA seq data generated in this study have been submitted to the NCBI Gene Expression Omnibus (https://www.ncbi.nlm.nih.gov/geo/query/acc.cgi?accGSE158832) under accession number GSE158832.

## Ethics Statement

The animal study was reviewed and approved by the Experimental Animal Health Ethics Committee of Beijing University of Chinese Medicine.

## Author Contributions

XG, JC, CB, QL, and TL conceived and designed the experiments. CB, RY, WX, and SL performed the experiments. CB, QL, and XP analyzed and interpreted the data. QL organized the original draft. XG, CB, and TL edited and reviewed the article. All authors have read, revised, and approved the final manuscript.

## Funding

This study has been supported by the Fundamental Research Funds for the Central Universities (No. 2019-JYB-JS-007), and China Postdoctoral Science Foundation (No. 2019M650593).

## Conflict of Interest

The authors declare that the research was conducted in the absence of any commercial or financial relationships that could be construed as a potential conflict of interest.

## Abbreviations

**Abbreviations:** A549, human endothelial lung cells; ACE2, angiotensin-converting enzyme 2; ALI, acute lung injury; ARDS, acute respiratory distress syndrome; BALB, Bagg’s albino mice; COVID-19, Corona Virus disease 2019; DEGs, differentially expressed genes; DPEP, D-pseudo-ephedrine; GL, glycyrrhizin; GO enrichment, gene ontology enrichment; H1N1, influenza A virus hemagglutinin; Hfl-1, human lung fibroblast cells; HSBD, Hua Shi Bai Du decoction; JHQG, Jin Hua Qing Gan granules; KM, Kunming mice; LC-ESI MS/MS, liquid chromatography-electrospray ionization tandem mass spectrometry; LEP, l-ephedrine; LHQW, Lian Hua Qing Wen capsules; Lico A, licochalcone A; LMEP, L-methylephedrin; LPS, lipopolysaccharide; MDCK, Madin-Darby canine kidney; MERS-CoV, Middle East Respiratory Syndrome Coronavirus; MXSG, Ma Xing Shi Gan decoction; PAMPs, pathogen-associated molecular patterns; PI3K/AKT, phosphatidylinositide 3-kinases/protein kinase B; PPAR, peroxisome proliferators-activated receptor; PRRs, pattern recognition receptors; QFPD, Qing Fei Pai Du decoction; SARS-CoV-2, Severe Acute Respiratory Syndrome Coronavirus 2; SD, Sprague-Dawley rats; T cell, T-lymphocyte; TCM, Traditional Chinese Medicine; TLRs, Toll-like receptors; WT, wild type mice; XFBD, Xuan Fei Bai Du decoction.
