# Supplementary material for: Deciphering the Pharmacological Mechanisms of Ma Xing Shi Gan Decoction against COVID-19 through Integrating Network Pharmacology and Experimental Exploration
Source: Front Pharmacol. 2020 Nov 26;11:581691. doi: 10.3389/fphar.2020.581691 (PMC7725906; doi:10.3389/fphar.2020.581691)
Supplement: Supplementary file 1 [file datasheet1.docx]

**Deciphering the Pharmacological Mechanisms of Ma Xing Shi Gan Decoction Against COVID-19 Through Integrating Network Pharmacology and Experimental Exploration**

Qianqian Li^†^, Chen Bai^†^, Ruocong Yang^†^, Weiying Xing, Xiaohan Pang, Siying Wu, Shaoyang Liu, Jianxin Chen^*^, Tiegang Liu^*^, Xiaohong Gu^*^

School of Traditional Chinese Medicine, Beijing University of Chinese Medicine, Beijing, China

***Correspondence:**

Xiaohong Gu, E-mail: guxh1003@126.com

Tiegang Liu, E-mail: [liutiegang2009@163.com](mailto:liutiegang2009@163.com)

Jianxin Chen, E-mail: [cjx@bucm.edu.cn](mailto:cjx@bucm.edu.cn)

†These authors have contributed equally to this work.

**Table S1** Compounds identified from MXSG by LC-ESI-MS/MS

| No. | Parent ion | Molecular Weight | Ionization model | Formula | Compounds | Class I |
| --- | --- | --- | --- | --- | --- | --- |
| 1 | 611.16 | 610.13 | [M+H]+ | C27H30O16 | Quercetin-3-O-glucoside-7-O-rhamnoside | Flavonoids |
| 2 | 515.12 | 516.11 | [M-H]- | C25H24O12 | 3,4-Dicaffeoylquinic acid | Phenolic acids |
| 3 | 282.28 | 281.25 | [M+H]+ | C18H35NO | Octadecenoic amide | Others |
| 4 | 169.02 | 170.02 | [M-H]- | C7H6O5 | Gallic acid | Flavonoids |
| 5 | 267.07 | 268.06 | [M-H]- | C16H12O4 | Formononetin | Flavonoids |
| 6 | 609.10 | 610.13 | [M-H]- | C27H30O16 | Rutin | Flavonoids |
| 7 | 447.09 | 448.08 | [M-H]- | C21H20O11 | Kaempferol 7-O-glucosdie | Flavonoids |
| 8 | 463.09 | 464.08 | [M-H]- | C21H20O12 | Isoquercitrin | Flavonoids |
| 9 | 146.10 | 145.05 | [M+H]+ | C9H7NO | Indole-3-carboxaldehyde | Alkaloids |
| 10 | 153.00 | 154.02 | [M-H]- | C7H6O4 | 2,5-Dihydroxybenzoic acid | Phenolic acids |
| 11 | 153.10 | 154.02 | [M-H]- | C7H6O4 | Protocatechuic acid | Flavonoids |
| 12 | 493.50 | 494.10 | [M-H]- | C26H22O10 | Salvianolic acid A | Phenolic acids |
| 13 | 255.00 | 256.06 | [M-H]- | C15H12O4 | Liquiritigenin | Flavonoids |
| 14 | 595.20 | 594.13 | [M+H]+ | C27H30O15 | Apigenin 6,8-C-diglucoside | Flavonoids |
| 15 | 177.03 | 178.02 | [M-H]- | C9H6O4 | Daphnetin | Lignans and Coumarins |
| 16 | 247.20 | 246.16 | [M+H]+ | C15H22N2O | Sophocarpine | Alkaloids |
| 17 | 249.20 | 248.17 | [M+H]+ | C15H24N2O | Matrine | Alkaloids |
| 18 | 271.10 | 272.09 | [M-H]- | C16H16O4 | Vestitol | Others |
| 19 | 433.00 | 434.10 | [M-H]- | C21H22O10 | Naringenin-7-O-glucoside | Flavonoids |
| 20 | 177.00 | 178.06 | [M-H]- | C10H10O3 | Trans-4-Hydroxycinnamic Acid Methyl Ester | Phenolic acids |
| 21 | 463.09 | 464.08 | [M-H]- | C21H20O12 | Gossypitrin | Flavonoids |
| 22 | 109.04 | 110.03 | [M-H]- | C6H6O2 | Pyrocatechol | Phenolic acids |
| 23 | 314.10 | 313.12 | [M+H]+ | C18H19NO4 | N-Feruloyltyramine | Alkaloids |
| 24 | 359.13 | 358.11 | [M+H]+ | C16H22O9 | Sweroside | Terpenoids |
| 25 | 456.15 | 457.14 | [M-H]- | C20H27NO11 | Amygdalin | Alkaloids |
| 26 | 565.15 | 564.12 | [M+H]+ | C26H28O14 | Schaftoside | Flavonoids |
| 27 | 609.18 | 608.15 | [M+H]+ | C28H32O15 | Diosmin | Flavonoids |
| 28 | 821.40 | 822.36 | [M-H]- | C42H62O16 | Monoammonium glycyrrhizinate | Terpenoids |
| 29 | 623.20 | 624.18 | [M-H]- | C29H36O15 | Forsythiaside A | Phenolic acids |
| 30 | 447.10 | 448.08 | [M-H]- | C21H20O11 | Astragalin | Flavonoids |
| 31 | 179.04 | 180.04 | [M-H]- | C9H8O4 | Caffeic acid | Phenolic acids |
| 32 | 359.15 | 358.13 | [M+H]+ | C20H22O6 | Matairesinol | Lignans and Coumarins |
| 33 | 317.07 | 316.05 | [M+H]+ | C16H12O7 | Tamarixetin | Flavonoids |
| 34 | 271.09 | 270.08 | [M+H]+ | C16H14O4 | Echinatin | Flavonoids |
| 35 | 339.10 | 338.14 | [M+H]+ | C21H22O4 | Licochalcone A | Flavonoids |
| 36 | 369.13 | 368.11 | [M+H]+ | C21H20O6 | Licorice coumarin | Lignans and Coumarins |
| 37 | 383.10 | 382.13 | [M+H]+ | C22H22O6 | Glycyrin | Lignans and Coumarins |
| 38 | 425.10 | 424.20 | [M+H]+ | C26H32O5 | Licoricidin | Others |
| 39 | 371.18 | 370.16 | [M+H]+ | C22H26O5 | Glyasperin D | Others |
| 40 | 469.00 | 470.34 | [M-H]- | C30H46O4 | Hypoglycyrrhizic acid(β) | Terpenoids |
| 41 | 468.90 | 470.34 | [M-H]- | C30H46O4 | Hypoglycyrrhizic acid(α) | Terpenoids |
| 42 | 367.11 | 366.10 | [M+H]+ | C21H18O6 | Neoglycyrol | Others |
| 43 | 391.40 | 392.18 | [M-H]- | C25H28O4 | Hispaglabridin A | Others |
| 44 | 337.14 | 338.14 | [M-H]- | C21H22O4 | 4'-O-Methylglabridin | Others |
| 45 | 136.10 | 135.05 | [M+H]+ | C5H5N5 | Aminopurine | Alkaloids |
| 46 | 411.10 | 410.16 | [M+H]+ | C23H26N2O5 | "N',N""-p-Coumaroyl-feruloyl putrescine" | Alkaloids |
| 47 | 337.10 | 338.09 | [M-H]- | C16H18O8 | 1-O-p-Coumaroyl quinic acid | Phenolic acids |
| 48 | 359.30 | 360.07 | [M-H]- | C18H16O8 | Rosmarinic acid | Phenolic acids |
| 49 | 431.11 | 432.09 | [M-H]- | C21H20O10 | Kaempferol 7-O-rhamnoside | Flavonoids |
| 50 | 255.06 | 254.05 | [M+H]+ | C15H10O4 | Daidzein | Flavonoids |
| 51 | 417.11 | 416.09 | [M+H]+ | C21H20O9 | Daidzein 7-O-glucoside(Daidzin) | Flavonoids |
| 52 | 353.00 | 354.08 | [M-H]- | C16H18O9 | Neochlorogenic acid(5-O-Caffeoylquinic acid) | Phenolic acids |
| 53 | 257.00 | 256.06 | [M+H]+ | C15H12O4 | Isoliquiritigenin | Flavonoids |
| 54 | 579.00 | 578.14 | [M+H]+ | C27H30O14 | Vitexin 2''-O-β-L-rhamnoside | Flavonoids |
| 55 | 285.00 | 284.06 | [M+H]+ | C16H12O5 | Calycosin | Flavonoids |
| 56 | 285.00 | 284.06 | [M+H]+ | C16H12O5 | Biochanin A | Flavonoids |
| 57 | 271.00 | 270.04 | [M+H]+ | C15H10O5 | 6-Hydroxydaidzein | Flavonoids |
| 58 | 447.12 | 446.10 | [M+H]+ | C22H22O10 | Sissotrin | Flavonoids |
| 59 | 273.07 | 272.06 | [M+H]+ | C15H12O5 | Butin | Flavonoids |
| 60 | 431.13 | 430.11 | [M+H]+ | C22H22O9 | Formononetin 7-O-glucoside(Ononin) | Flavonoids |
| 61 | 519.16 | 520.17 | [M-H]- | C26H32O11 | Terpineol monoglucoside | Lignans and Coumarins |
| 62 | 515.12 | 516.11 | [M-H]- | C25H24O12 | Isochlorogenic acid A | Phenolic acids |
| 63 | 325.09 | 326.09 | [M-H]- | C15H18O8 | 1-O-[(E)-p-Cumaroyl]-β-D-glucopyranose | Phenolic acids |
| 64 | 337.09 | 338.09 | [M-H]- | C16H18O8 | 3-O-(E)-p-Coumaroyl quinic acid | Phenolic acids |
| 65 | 179.03 | 180.04 | [M-H]- | C9H8O4 | Sorbic acid | Phenolic acids |
| 66 | 371.10 | 372.09 | [M-H]- | C16H20O10 | Trihydroxycinnamoylquinic acid | Phenolic acids |
| 67 | 623.20 | 624.18 | [M-H]- | C29H36O15 | Verbascoside | Phenolic acids |
| 68 | 433.10 | 432.09 | [M+H]+ | C21H20O10 | Apigenin-8-C-glucoside | Flavonoids |
| 69 | 797.46 | 796.41 | [M+H]+ | C42H68O14 | Soyasaponin βb’ | Terpenoids |
| 70 | 913.51 | 912.45 | [M+H]+ | C47H76O17 | Soyasaponin βc | Terpenoids |
| 71 | 943.52 | 942.46 | [M+H]+ | C48H78O18 | Soyasaponin βb(Soyasaponin I) | Terpenoids |
| 72 | 957.49 | 956.44 | [M+H]+ | C48H76O19 | Soyasaponin βd | Terpenoids |
| 73 | 959.51 | 958.46 | [M+H]+ | C48H78O19 | Soyasaponin βa | Terpenoids |
| 74 | 339.10 | 338.09 | [M+H]+ | C16H18O8 | Trans-3-O-p-coumaric quinic acid | Phenolic acids |
| 75 | 337.11 | 336.09 | [M+H]+ | C20H16O5 | Glabrone | Flavonoids |
| 76 | 339.12 | 338.10 | [M+H]+ | C20H18O5 | Eurycarpin A | Flavonoids |
| 77 | 339.12 | 338.10 | [M+H]+ | C20H18O5 | Licoflavone C | Flavonoids |
| 78 | 369.13 | 368.11 | [M+H]+ | C21H20O6 | Glisoflavone | Flavonoids |
| 79 | 551.18 | 550.14 | [M+H]+ | C26H30O13 | Liquiritin apioside | Flavonoids |
| 80 | 823.41 | 822.36 | [M+H]+ | C42H62O16 | Isoglycyrrhizic acid | Terpenoids |
| 81 | 447.12 | 446.10 | [M+H]+ | C22H22O10 | 3'-Methoxydaidzin | Flavonoids |
| 82 | 285.07 | 284.06 | [M+H]+ | C16H12O5 | 3'-Methoxydaidzein | Flavonoids |
| 83 | 417.12 | 416.09 | [M+H]+ | C21H20O9 | Daidzein-4'-glucoside | Flavonoids |
| 84 | 355.10 | 354.08 | [M+H]+ | C16H18O9 | Chlorogenic Acid | Phenolic acids |
| 85 | 447.12 | 446.10 | [M+H]+ | C22H22O10 | Calycosin-7-glucoside | Flavonoids |
| 86 | 593.18 | 592.15 | [M+H]+ | C28H32O14 | Acacetin-7-O-rutinoside | Flavonoids |
| 87 | 449.10 | 448.08 | [M+H]+ | C21H20O11 | Rhamnone-2-O-B-D-Glucopyranoside from Italy | Quinones |
| 88 | 563.17 | 562.14 | [M+H]+ | C27H30O13 | Kushenol O | Flavonoids |
| 89 | 152.11 | 151.09 | [M+H]+ | C9H13NO | Norephedrine | Alkaloids |
| 90 | 166.12 | 165.11 | [M+H]+ | C10H15NO | Ephedrine | Alkaloids |
| 91 | 166.12 | 165.11 | [M+H]+ | C10H15NO | Pseudoephedrine | Alkaloids |
| 92 | 180.14 | 179.12 | [M+H]+ | C11H17NO | Methylephedrine | Alkaloids |
| 93 | 342.20 | 342.15 | [M]+ | C20H24NO4+ | Magnoflorine | Alkaloids |
| 94 | 314.10 | 313.12 | [M+H]+ | C18H19NO4 | Methoxy-N-Caffeoyltyramine | Alkaloids |
| 95 | 314.14 | 313.13 | [M+H]+ | C18H19NO4 | N-Cis-feruloyltyramine | Alkaloids |
| 96 | 478.29 | 477.25 | [M+H]+ | C23H44NO7P | 3-{(2-Aminoethoxy)(hydroxy)phosphoryl]oxy}-2-12-octadecadienoate | Alkaloids |
| 97 | 120.08 | 119.07 | [M+H]+ | C8H9N | N-Benzylmethylene isomethylamine | Alkaloids |

**Table S2.** The corresponding target information of 54 compounds detected

| No. | Gene symbol | No. | Gene symbol | No. | Gene symbol | No. | Gene symbol |
| --- | --- | --- | --- | --- | --- | --- | --- |
| 1 | ACHE | 52 | CHRNA2 | 103 | ICAM1 | 154 | PIM1 |
| 2 | ADH1C | 53 | COL7A1 | 104 | IGF1 | 155 | PKIA |
| 3 | ADRA1A | 54 | COMT | 105 | IGF1R | 156 | PLAU |
| 4 | ADRA1B | 55 | CPT1A | 106 | IGF2 | 157 | POR |
| 5 | ADRA1D | 56 | CTNNA1 | 107 | IGHG1 | 158 | PPARD |
| 6 | ADRA2A | 57 | CTNNB1 | 108 | IL10 | 159 | PPARG |
| 7 | ADRA2B | 58 | CTRB1 | 109 | IL1B | 160 | PPARGC1B |
| 8 | ADRA2C | 59 | CYP17A1 | 110 | IL4 | 161 | PRKACA |
| 9 | ADRB1 | 60 | CYP1A1 | 111 | IL6 | 162 | PRKCA |
| 10 | ADRB2 | 61 | CYP21A2 | 112 | IL8 | 163 | PRKCB |
| 11 | AHR | 62 | CYP2E1 | 113 | INS | 164 | PRKCG |
| 12 | AKR1B1 | 63 | CYP3A4 | 114 | ITGB2 | 165 | PRKCZ |
| 13 | AKR1C3 | 64 | CYP3A43 | 115 | JAK2 | 166 | PRSS1 |
| 14 | AKT1 | 65 | DIO1 | 116 | JUN | 167 | PRSS3 |
| 15 | ALDH1B1 | 66 | DPEP1 | 117 | JUP | 168 | PTGS1 |
| 16 | ALDH2 | 67 | DPP4 | 118 | KCNH2 | 169 | PTGS2 |
| 17 | ALOX5 | 68 | DRD1 | 119 | KCNMA1 | 170 | PTPN1 |
| 18 | APOB | 69 | ECE1 | 120 | KDR | 171 | RAC1 |
| 19 | AR | 70 | EIF6 | 121 | LARS | 172 | RAD51 |
| 20 | ATMIN | 71 | EP300 | 122 | LARS1 | 173 | RAD51B |
| 21 | ATP5B | 72 | EPHX1 | 123 | LDLR | 174 | RAF1 |
| 22 | ATP5F1B | 73 | ESR | 124 | LTA4H | 175 | RB1 |
| 23 | B4GALT4 | 74 | ESR1 | 125 | LYZ | 176 | RBM45 |
| 24 | BACE1 | 75 | ESR2 | 126 | MAOA | 177 | RELA |
| 25 | BAP1 | 76 | F10 | 127 | MAOB | 178 | RHOA |
| 26 | BARD1 | 77 | F11R | 128 | MAPK1 | 179 | RXRA |
| 27 | BAX | 78 | F2 | 129 | MAPK14 | 180 | SCN5A |
| 28 | BCL2 | 79 | F7 | 130 | MAPK3 | 181 | SELE |
| 29 | BTK | 80 | FASLG | 131 | MGAM | 182 | SELP |
| 30 | C5AR1 | 81 | FASN | 132 | MGST1 | 183 | SIRT1 |
| 31 | CA2 | 82 | FCER2 | 133 | MKI67 | 184 | SLC2A1 |
| 32 | CALM1 | 83 | FOS | 134 | MPO | 185 | SLC6A2 |
| 33 | CALM3 | 84 | FOSL2 | 135 | MT2A | 186 | SLC6A20 |
| 34 | CASP3 | 85 | GAA | 136 | MT-ND6 | 187 | SLC6A3 |
| 35 | CASP9 | 86 | GABBR1 | 137 | MTTP | 188 | SLC6A4 |
| 36 | CAT | 87 | GABRA2 | 138 | MYB | 189 | SOD1 |
| 37 | CAV1 | 88 | GADD45A | 139 | MYC | 190 | SOD2 |
| 38 | CCNA2 | 89 | GFAP | 140 | NCOA1 | 191 | STAT1 |
| 39 | CCND1 | 90 | GH1 | 141 | NCOA2 | 192 | STAT3 |
| 40 | CD5L | 91 | GHR | 142 | NFE2L2 | 193 | SULT1A1 |
| 41 | CDC25A | 92 | GJA1 | 143 | NOS2 | 194 | TBXA2R |
| 42 | CDK2 | 93 | GSK3B | 144 | NOS3 | 195 | TFF1 |
| 43 | CDK4 | 94 | GSTP1 | 145 | NQO2 | 196 | TGFB1I1 |
| 44 | CDKN1A | 95 | HAS2 | 146 | NR1I2 | 197 | TNF |
| 45 | CHEK1 | 96 | HERC5 | 147 | NR3C1 | 198 | TOP2B |
| 46 | CHKB | 97 | HMGCR | 148 | OPRD1 | 199 | TP53 |
| 47 | CHRM1 | 98 | HSD11B2 | 149 | OPRM1 | 200 | TRPM2 |
| 48 | CHRM2 | 99 | HSD3B1 | 150 | P4HB | 201 | TYR |
| 49 | CHRM3 | 100 | HSD3B2 | 151 | PDE3A | 202 | VCAM1 |
| 50 | CHRM4 | 101 | HSP90 | 152 | PGR | 203 | VEGFA |
| 51 | CHRM5 | 102 | HTR2A | 153 | PIK3CG |  |  |

**Table S3** Formulation, effects and clinical features of MXSG and its extended formula

| Drug | Effects | Formulation (Latin name) | Clinical features |
| --- | --- | --- | --- |
| **MXSG** | Clearing heat and preventing asthma, dispersing lung and relieving cough | *Herba Ephedrae, Semen armeniacae amarum, Gypsum fibrosum and Radix Glycyrrhizae* | Warm Heat Pathogen Congesting the Lung: fever, cough with dyspnea, thirst, nasal flaring, sweating or anhidrosis, tongue coating thin white or yellow, slippery pulse and rapid. |
| ***LHQW*** | Clearing pestilence and detoxication, dispersing lung and dispersing heat | *Fructus Forsythiae, Flos Lonicerae Japonicae, Herba Ephedrae, Radix Et Rhizoma Rhei, Semen Mentholum, Armeniacae Amarum, Gypsum Fibrosum, Radix Isatidis, Herba Houttuyniae, Rhizoma Dryopteridis Crassirhizomatis, Herba Pogostemonis, Radix Et Rhizoma Rhodiolae Crenulatae, and Radix Glycyrrhizae* | Influenza belongs to syndrome of noxious heat attacking lung, including fever or hyperthermia, aversion to cold, muscles aches, nasal congestion and rhinorrhea, cough, headache, pharyngeal dryness and pharyngalgia, tongue texture with red, tongue coating with yellow or yellow-greasy, etc. |
| **JHQG** | Dispelling wind and dispersing lung, clearing heat and detoxication | *Flos Lonicerae Japonicae, Gypsum Fibrosum, Herba Ephedrae, Mentholum, Semen Armeniacae Amarum, Fructus Forsythiae, Fructus Arctii, Radix Scutellariae, Herba Artemisiae Annuae, Bulbus Fritillariae Thunbergii, Rhizoma Anemarrhenae, and Radix Glycyrrhizae* | Mild symptom of simplex influenza, the syndrome differentiation of TCM belongs to wind-heat attacking the lung syndromes, including fever, headache, body aches, pharyngalgia, cough, aversion to wind or cold, nasal congestion and rhinorrhea, tongue texture with red, tongue coating with yellow-thin, rapid pulse. |
| **QFPD** | Dispersing lung and relieving cough, clearing heat and resolving dampness, repelling filth detoxication | *Herba Ephedrae, Radix Glycyrrhizae, Ramulus Cinmomi, Polyporus umbellatus, Semen Armeniacae Amarum, Gypsum fibrosum, Rhizoma Alismatis, Atractylodis Macrocephalae Rhizoma, Stellariae Radix, Radix Asteris, Poria, Radix Scutellariae, Pinelliae Rhizoma Praeparatum cum Zingibere, Rhizoma Zingiberis Recens, Flos farfarae, Herba Asari, Rhizoma Belamcandae, Rhizoma Dioscoreae, Fructus Aurantii Immaturus, Pericarpium Citri Reticulatae, and Pogostemon Cablin Benth* | Based on the clinical observations made by clinicians across different regions, this is a basic Chinese herbal medicine formula applies to mild cases, moderate cases, and severe cases. It may also apply to critical cases, depending on the condition of individual patients. Where appropriate, medical professionals may choose to prescribe other formulae introduced in the subsequent sections of this article, based on the TCM diagnosis of patients. |
| **XFBD** | Pungent-cool and ventilate-discharge lung, clearing lung-heat and preventing asthma | *Gypsum Fibrosum, Semen Armeniacae Amarum, Herba Ephedrae, Pogostemi Herba, Coicis Semen Crudum, Atractylodes Rhizome, Herba Artemisiae Annuae, Rhizome Polygoni Cuspidate, Exocarpium Citri Grandis, Herba Verbanae, Rhizome Phragmitis, Semen Lepidii, and Radix Glycyrrhizae* | Fever, coughing with small amounts of sputum or coughing with yellow sputum, chest discomfort, shortness of breath, abdominal distension, and constipation. Dark red and enlarged tongue. Yellow and slimy tongue fur, or yellow and dry tongue fur. Slippery and rapid pulse, or string-like and soggy pulse. |
| **HSBD** | Detoxication and resolving dampness, clearing heat and preventing asthma | *Gypsum Fibrosum, Herba Ephedrae, Officinal Magnolia Bark, Semen Armeniacae Amarum, Radix Glycyrrhizae, Herba Pogostemonis, Fructus Tsaoko, Rhizmrn Atractylodis, Rhizome Pinelliae, Semen Lepidii, Indian Bread, Rhubarb, Radix Astragali, and Radix Paeoniae Rubra* | Fever, flushed face, coughing with small amounts of sticky yellow sputum or with blood, panting, shortness of breath, fatigue, dry mouth with bitter taste and sticky feeling in the mouth, loss of appetite, nausea, ungratifying defecation, reddish urine with reduced amount. Red tongue. Yellow and slimy tongue fur. Slippery and rapid pulse. |

Note: Information is from corresponding drug labels and the latest Guideline for the Diagnosis and Treatment of COVID-19 promulgated by National Health Commission of the People’s Republic of China.

**Table S4** Composition of Ma Xing Shi Gao decoction (MXSG) and the characteristics of each constituent drugs.

| **Drug name** | **Scientific name (Family)** | **Medicinal part** | **Weight (g) in MXSG** | | **Medicinal efficacies** | **Role in the prescription** |
| --- | --- | --- | --- | --- | --- | --- |
| Ephedra sinica Stapf | Ephedraceae | Stem | | 8 | Lung, bladder | Monarch |
| Prunus armeniaca L. | *Rosaceae* | seed | | 6 | Lung, large intestine | Assistant |
| Glycyrrhiza glabra L. | *Fabaceae* | Rhizome | | 4 | Spleen, stomach，lung | Messenger |
| Gypsum fibrosum | / | Gypsum(mineral) | | 16 | Lung, stomach | Minister |


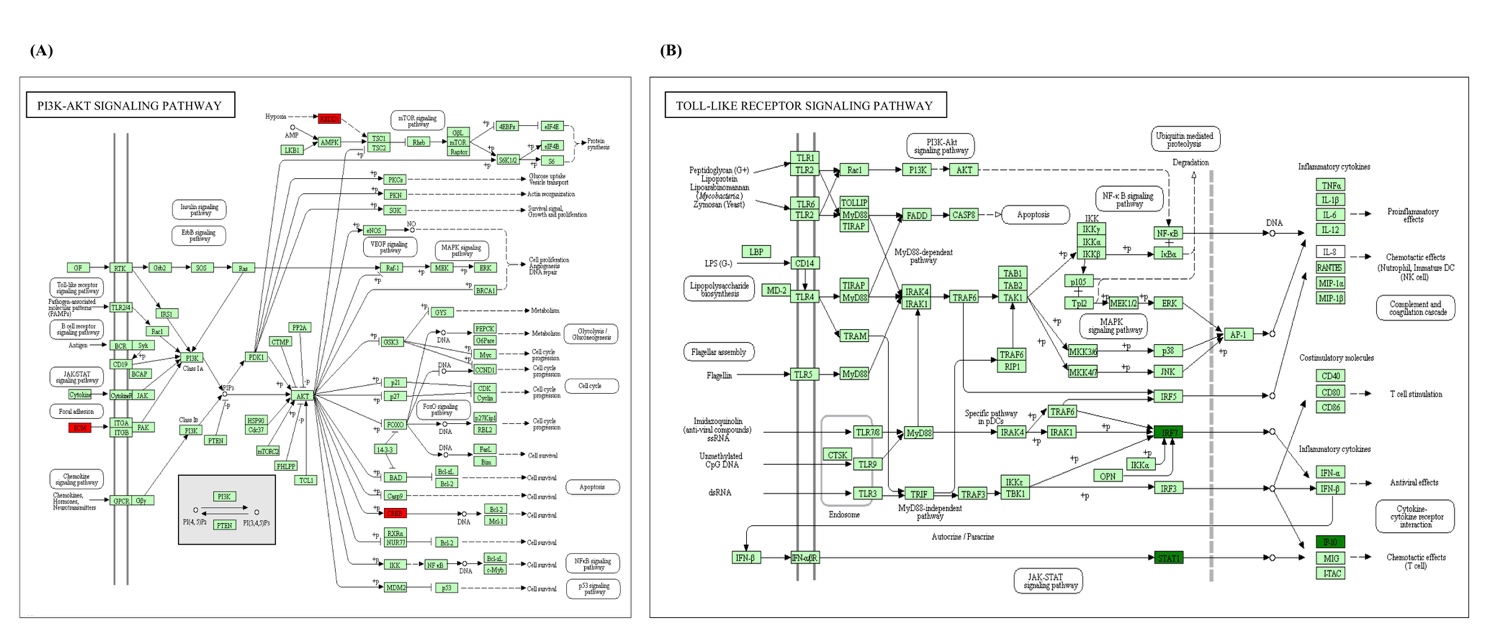


**Fig. S1** MXSG-related regulatory pathways of lung tissue transcriptome. (A) PI3K-Akt signaling pathway; (B) Toll-like Receptor signaling pathway. Red contents in the figure indicate up-regulated genes, green indicates down-regulated genes.
